# Thymoquinone improves folliculogenesis, sexual hormones, gene expression of apoptotic markers and antioxidant enzymes in polycystic ovary syndrome rat model

**DOI:** 10.1002/vms3.958

**Published:** 2022-09-14

**Authors:** Sanaz Alaee, Maryam Mirani, Zahra Derakhshan, Farhad Koohpeyma, Azizollah Bakhtari

**Affiliations:** ^1^ Department of Reproductive Biology School of Advanced Medical Sciences and Technologies Shiraz University of Medical Sciences Shiraz Iran; ^2^ Stem Cells Technology Research Center Shiraz University of Medical Sciences Shiraz Iran; ^3^ Endocrine and Metabolism Research Center Shiraz University of Medical Sciences Shiraz Iran

**Keywords:** Bax, Bcl2, FSH, LH, testosterone

## Abstract

**Background:**

Nowadays, polycystic ovary syndrome (PCOS) is a prevalent cause of infertility affecting women of reproductive age around the world. Thymoquinone is a natural antioxidant, derived from *Nigella sativa*.

**Objectives:**

The current study aimed to evaluate the protective effects of thymoquinone on the detrimental effects of PCOS rats induced with letrozole.

**Methods:**

Thirty‐two female rats were randomly divided into four groups: (1) Control, (2) PCOS, (3) PCOS+5 mg/kg thymoquinone and (4) PCOS+10 mg/kg thymoquinone. Thymoquinone was administered every 3 days for 30 days. Ovaries were histopathologically and stereologically examined, and antioxidant and apoptotic enzymes gene expression in ovaries and sex hormones in serum were measured.

**Results:**

The number of unilaminar, multilaminar, antral, and graffian follicles, volume density of corpus luteum (*p* < 0.01), and *GPx1* gene expression in ovaries and level of FSH in the blood increased in both thymoquinone groups when compared to untreated PCOS (*p* < 0.05). Ovaries in thymoquinone groups showed a significant reduction in the number of atretic follicles, ovary weight and volume, volume density of cortex and ovarian cysts, *Bax* gene expression (*p* < 0.01) and *Bax/Bcl2* ratio as well as levels of luteinizing hormone (LH), LH/FSH ratio and testosterone (*p* < 0.05) in the blood of female rats when compared to PCOS group. Administration of thymoquinone restored the most detrimental effects of PCOS on ovaries (*p* < 0.01) and sexual hormones (*p* < 0.05) in rats.

**Conclusions:**

These data suggest that thymoquinone has improved effects on ovarian function in the PCOS rat model. Therefore, thymoquinone might be useful as a protective agent and adjunct treatment in PCOS patients.

## Graphical abstract

Thymoquinone improved levels of sexual hormones in blood, and had putative beneficial on ovarian function by amelioration of folliculogenesis and oxidative stress in the ovary of PCOS rats. Therefore, thymoquinone might be useful as a protective agent and adjunct treatment in PCOS patients.

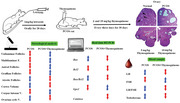

## 1 INTRODUCTION

Polycystic ovary syndrome (PCOS) is an endocrine condition marked by hyperandrogenism, high oxidative stress, and in some cases, obesity, irregular menstrual cycles, insulin resistance, and oligomenorrhea or anovulation, among other symptoms (Victor et al., 2016). Alterations in gonadotropin secretion caused by PCOS have a direct impact on the ovary. Enhanced gonadotropin‐releasing hormone (GnRH) pulsatility, increased luteinizing hormone (LH) and reduced follicle‐stimulating hormone (FSH) produce anovulation and androgen abundance (Dumesic & Richards, 2013). Promotion of androgen activity in women with PCOS leads to changes in gonadotropin‐triggered oestrogen and progesterone synthesis in the ovarian follicle (Lo et al., 2017). Several morphological changes occur in PCOS, including increased ovarian cortical thickness, the existence of numerous follicular cysts and stromal hyperplasia, all of which lead to folliculogenesis disruption (Dunaif, 2012).

In PCOS, the generation of oxidative stress appears to be multifactorial. An adverse redox status is caused by genetic abnormalities, epigenetic alterations during the syndrome's developmental course and the basic contribution of environmental factors (Papalou et al., 2016). Increased reactive oxygen species (ROS) levels cause DNA damage, endothelial destruction, and granulosa cell apoptosis and ovarian damage (Majdi Seghinsara et al., 2019; Shokoohi et al., 2019; Shokri et al., 2019).

Chemical drugs, such as metformin, have been associated with serious side effects that have a detrimental impact on patients’ quality of life (Murri et al., 2018). From the past to the present, herbal plants and their natural antioxidants with pharmacological potential were used as an appropriate treatment strategy for patients (Venkateswara Rao et al., 2017). Various types of research are undertaken using herbal plants and their extraction for the treatment and management of male fertility (Adewale et al., 2021; Emokpae & Olaode, 2021; Feyisike et al., 2020; Ojatula, 2020; Oyetunji et al., 2021) and PCOS (Sadeghi Ataabadi et al., 2017; Sherafatmanesh et al., 2020). These plants are becoming increasingly popular in both developing and developed countries as a result of their accessibility, lack of adverse effects and ease of use (Hasani‐Ranjbar & Larijani, 2014).

*Nigella sativa* (*N. sativa*), commonly known as black seed or black cumin, is a plant that has been used to treat a variety of ailments all over the world for thousands of years. It is a Mediterranean annual herbal plant from the Ranunculaceae family. Fixed and essential oils, proteins, alkaloids and saponin can all be found in this seed. Thymoquinone, a key component of the essential oil, has been demonstrated to be responsible for much of the biological activity of *N. sativa* (Ali & Blunden, 2003), such as anti‐oxidative, anti‐inflammatory, anti‐tumour and neuroprotective effects (Ali et al., 2020; Darakhshan et al., 2015).

Given these previously published findings, in this study, we appraised the amelioration effects of thymoquinone as an antioxidant compound on reproductive hormones, folliculogenesis, number of each follicle, the volume of different parts of ovary tissue, and gene expression of apoptotic markers and antioxidant enzymes in a rat model of letrozole‐induced PCOS.

## 2 MATERIALS AND METHODS

### 2.1 Animals

Thirty‐two mature Wistar albino female rats (203±7 g) were obtained from the animal house of Shiraz University of Medical Sciences, Shiraz, Iran. Animals were kept in cages at standard room temperature (22±2 C) with constant humidity (40–50%) and 12 h/12 h light/dark cycle with free access to standard diet and water before the experiment. Daily vaginal smears were carried out for estrous cycle evaluation in rats. Animals with two normal estrus cycles were weighed and allocated into four groups as below:

Group I (Control): Received 1 ml distilled water orally for 28 days; Group II (PCOS): Received 1 mg/kg letrozole (Femara, Novartis, Basel, Switzerland) orally for 28 days (Sadeghi Ataabadi et al., 2017; Sherafatmanesh et al., 2020); Group III (L.D.): Received letrozole orally for 28 days and then intraperitoneal received 5 mg/kg thymoquinone every 3 days for 30 days; and Group IV (H.D.): Received letrozole orally for 28 days and then intraperitoneal received 10 mg/kg thymoquinone every 3 days for 30 days. Letrozole had been dissolved in normal saline.

To confirm the induction of PCOS, a microscopic examination of the collected vaginal smears was performed to observe the persistent estrus phase. Furthermore, induction of PCOS was approved by observation of a high number of ovarian cysts in ovarian sections via haematoxylin and eosin (H&E) staining. After the final administration of mentioned materials in each group, rats were killed and a blood sample was taken from the heart for hormonal analysis. In addition, the ovarian tissues of all rats were collected and weighed. Each left ovary was preserved in buffer formalin for histological evaluation by unbiased stereology method and each right ovary was placed in a 2 ml microtube (RNase and DNase free, Greiner Bio‐One, Germany) and maintained at –70°C until quantitative real‐time PCR evaluation for apoptotic markers and antioxidant enzymes.

### 2.2 Hormonal assay

At the end of the experiment, serum samples were isolated by centrifugation at 6000 rpm for 5 min. Serum LH, FSH, LH/FSH ratio (Hangzhao Eastbiopharm Co., Ltd., Hangzhou, China) and testosterone (Padtan Elm Company, Tehran, Iran) levels were measured by ELISA kits according to the manufacturer's protocol.

### 2.3 Tissue preparation, histological analysis and stereological measurements

The left ovaries of rats were fixed in a 4% buffered formalin solution. After tissue processing, the ovaries were separately placed in cylindrical paraffin blocks. Serial sections of ovaries with a different thickness (5 and 20 μm) were obtained using a microtome and stained with H&E (Merck company, Germany) method to estimate the volume of cortex, medulla, corpus luteum and ovarian cysts, and counting the number of primordial follicles, primary follicles, secondary follicles, Graafian follicles, atretic follicles, corpus luteum and cysts.

**FIGURE 1:**
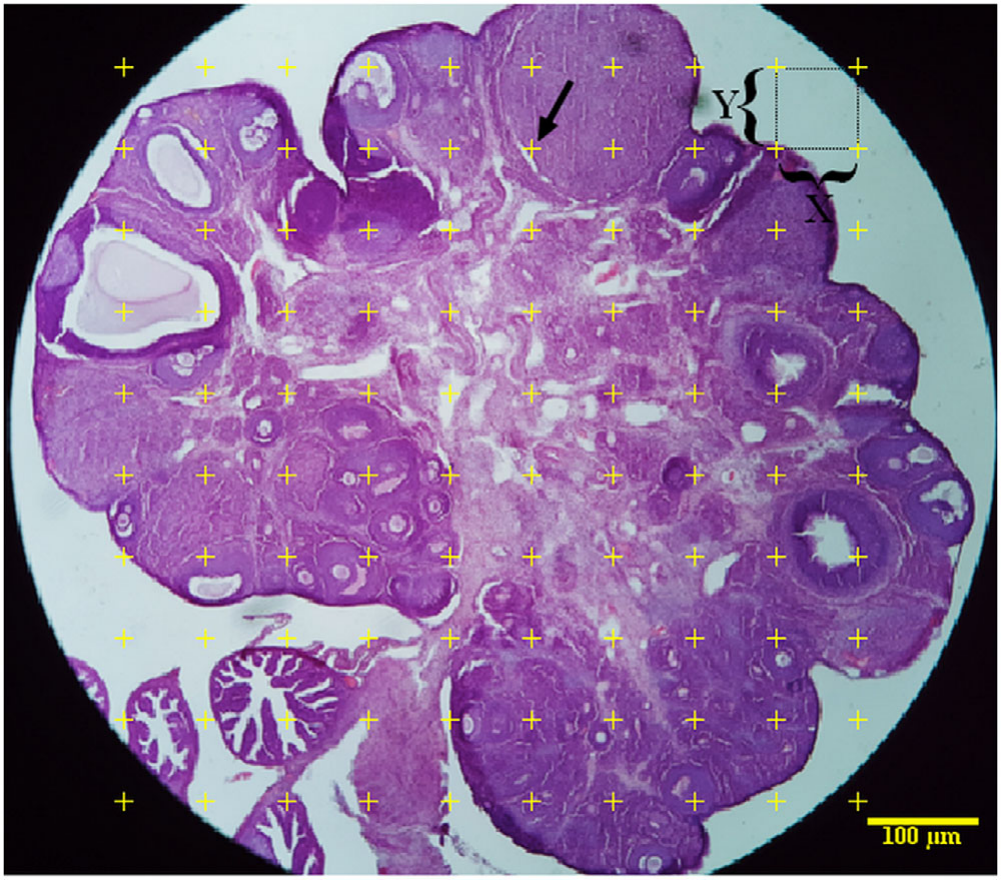
Approximation of the ovarian volume and volume density of the cortex, medulla, corpus luteum and ovarian cysts using the point‐counting method. The countable points are hitting the cortex, medulla, corpus luteum and ovarian cysts at the right upper corner of the cross (arrow).

The total volume of the ovary was calculated using the Cavalieri method. To create isotropic uniform random sections, the orientator approach was utilized (Howard & Reed, 2004). A total of 8–12 slides from each ovary were chosen by this method. A counting probe was placed on the images at random, and the total number of points hitting the sections was counted (Figure 1). The total volume of the ovary was estimated using the following formulas:

(1) $$V_{total\;ovary=}\sum_{i\;=\;1}^{n}p\times a\left(p\right)\;\times t$$

‘$\sum_{i\;=\;1}^{n}p$’ was the total number of superimposed points on the image, ‘*a*(*p*)’ was the area corresponding to each point and ‘*t*’ was the distance between the sample sections.

Volume density of cortex, medulla, corpus luteum and ovarian cysts was estimated on 5 μm thickness sections through the point‐counting method and using Delesse's formula (Noorafshan et al., 2013) (Figure 1):

(2) $$Vv\left(structure\right)=\sum_{i=1}^{n}p\;\left(structure\right)/\sum_{i=1}^{n}\left(reference\right)$$

‘$\;\sum_{i\;=\;1}^{n}p(structure$)’ was the number of the test points falling on the cortex, medulla, corpus luteum and ovarian cysts, and ‘$\sum_{i\;=\;1}^{n}p$ (reference)’ was the total number of points that hit in the ovarian sections. The absolute volume of cortex, medulla, corpus luteum and ovarian cysts was calculated using the below formula (Noorafshan et al., 2015):

(3) $$V\left(structure\right)=V\left(ovary\right)\;\times Vv\left(structure\right)$$

**FIGURE 2:**
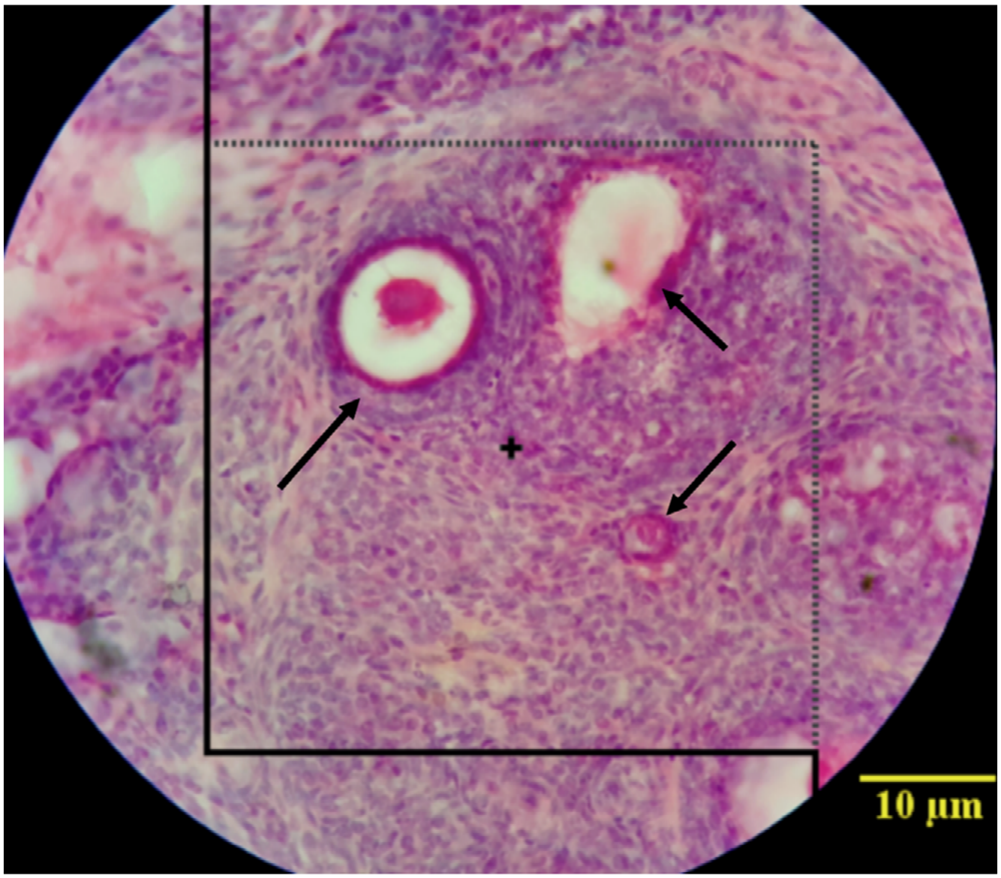
Estimation of the number of follicles using the counting frame; arrows indicate follicles types.

The number of follicles was determined on 20 μm thickness sections using an optical Disector method (Figure 2). The numerical density (*Nv*) or the number of follicles in the unit was calculated by the following formula:

(4) $$Nv=\frac{\sum_{i=1}^{n}Q}{\sum_{i=1}^{n}P\times \;h\;\times \;\left(\frac{a}{f}\right)}\times \frac{t}{BA}\;$$

‘$\sum_{i=1}^{n}Q$’ was the number of the follicles counted across all Disectors, ‘*h*’ was the optical Disector's height, ‘*a*/*f*’ was the area of the counting frame, ‘$\sum_{i=1}^{n}P$’ was the total number of the counted frames, ‘*BA*’ was the microtome's setting for cutting the paraffin block and ‘*t*’ was the mean of the final section thickness (Samare‐Najaf et al., 2020). The following formula was used to calculate the total number of follicles: *N*_(Follicles)_ = *N_V_*_(follicles/ovary)_ × *V*_(ovary)_

### 2.4 RNA extraction, cDNA synthesis and quantitative real‐time RT‐PCR

Total RNA was extracted from ovaries using the RNX plus (Cinnagen, Iran) following the manufacturer's instructions. First‐strand cDNA synthesis was carried out using the QuantiTect Reverse Transcription Kit (Qiagen, Germany) according to the manufacturer's instructions. Real‐time RT‐PCR was performed using an ABI Prism 7500 Sequence Detection System (Applied Biosystems, Foster City, CA, USA). The PCR amplification was carried out in a final volume of 25 μl, including 1 μl of the cDNA template, 1 μl of each primer (10 pmol/μl) and 12.5 μl of RealQ Plus 2x Master Mix Green Low ROX (Ampliqon, Odense, Denmark). Beta‐actin (*Actb*) was used as a reference gene for normalization of the target gene dosage level. Genes expression of glutathione peroxidase 1 (*GPx1*), superoxide dismutase 1 (*Sod1*) and catalase (*Cat*) as main antioxidant enzymes, and *Bcl2* (apoptotic inhibitor) and *Bax* (apoptotic activator) in ovaries were analysed. Samples were analysed using the 2^−∆∆Ct^ method. The primers used for RT‐PCR are listed in Table 1.

**TABLE 1:** Details of primers used for quantitative real‐time RT‐PCR

Gene symbol	Nucleotide sequences (5′–3′)	Fragment size (bp)	Accession number
*GPx1*	F: CAGGAGAATGGCAAGAATGAAGAG	136	NM_030826.4
	R: GGAAGGTAAAGAGCGGGTGA		
*Sod1*	F: TCCACGAGAAACAAGATGACT	92	NM_017050.1
	R: AATCACACCACAAGCCAAG		
*Cat*	F: TTTCCCACAAGGTCCCAGTTA	115	NM_012520.2
	R: AATTGCGTTCTTAGGCTTCTCAG		
*Bcl2*	F: ATGCGACCTCTGTTTGATTT	115	NM_016993.2
	R: TCTGCTGACCTCACTTGT		
*Bax*	F: GCTACAGGGTTTCATCCA	136	NM_017059.2
	R: GTCCAGTTCATCGCCAAT		
*Actb*	F: CCACACCCGCCACCAGTTCG	138	NM_031144.3
	R: CTAGGGCGGCCCACGATGGA		

### 2.5 Statistical analysis

Hormone level, folliculogenesis and real‐time RT‐PCR data were analysed by one‐way ANOVA followed by Tukey's multiple comparison test using the GraphPad Prism version 6 (San Diego, CA, USA). Data were expressed as Mean± Standard Deviation (SD). Differences were considered as significant at *p* < 0.05.

## 3 RESULTS

### 3.1 Developmental stages of folliculogenesis

**FIGURE 3:**
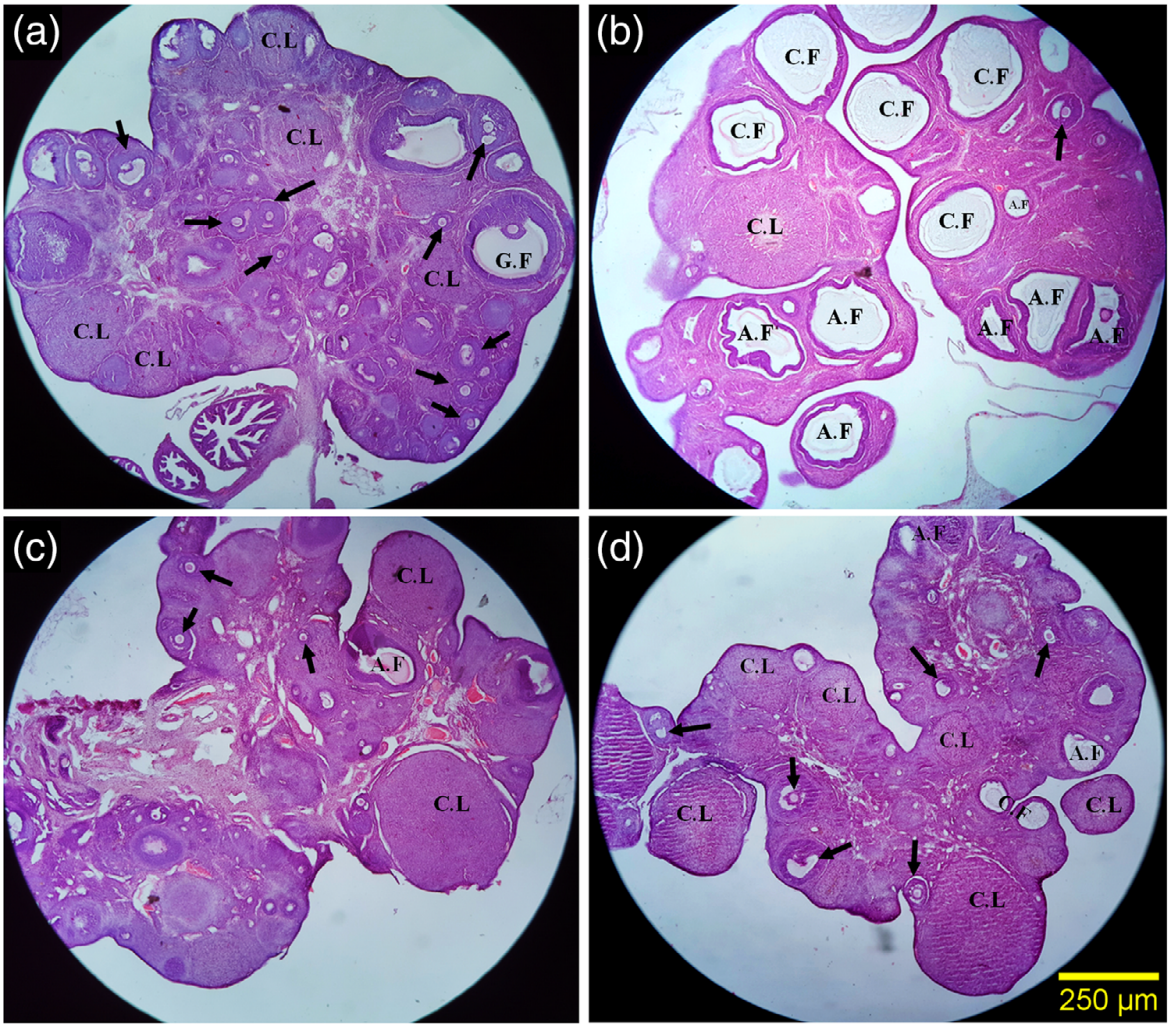
Comparison of the microscopic images of the ovaries in different steps of intermediation. Control group (a): The different developmental stages of folliculogenesis were normal in the healthy control groups, PCOS group (b): The PCOS group showed a significant increase in the number of ovarian cyst and atretic follicles, PCOS+ 5 mg/kg thymoquinone group (c) and PCOS+ 10 mg/kg thymoquinone group (d): In these groups, a significant reduction in the number of ovarian cysts and atretic follicle were observed along with the higher volume of the corpus luteum and healthy follicles. a–d: H&E staining with magnification at × 40. The arrow indicates normal follicles (antral and preantral follicles). Abbreviations: A.F, atretic follicles; C.F, cystic follicles; C.L, corpus luteum; G.F, graafian follicles.

**FIGURE 4:**
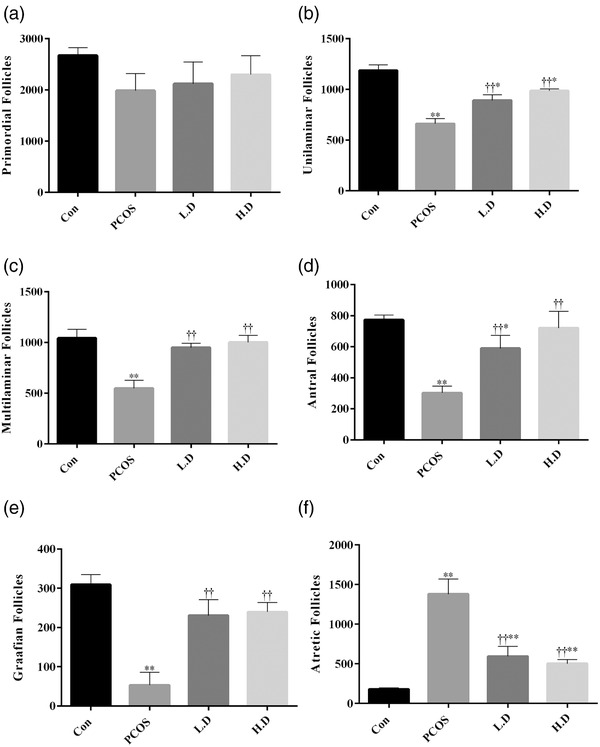
The number of follicles was determined on 20 μm thickness sections (*n* = 8). The results are shown as the Mean ± SD. Abbreviations: Con, control; H.D, PCOS + 10 mg/kg thymoquinone; L.D, PCOS + 5 mg/kg thymoquinone; PCOS, polycystic ovary syndrome. *p < 0.05 and **p < 0.01, Con group vs. PCOS, L.D and H.D. ††*p* < 0.01, PCOS vs. L.D and H.D groups.

The developmental stages of folliculogenesis were normal in the healthy control group. The number of primordial follicles showed no significant difference among groups. The number of unilaminar, multilaminar, antral and graffian follicles decreased significantly in the ovaries of PCOS rats compared to that of the control group (*p* < 0.01), and the number of mentioned follicles was significantly higher in PCOS ovaries of rats which received 5 and 10 mg/kg thymoquinone in comparison to ovaries of PCOS rats without any intervention (*p* < 0.01). However, 5 and 10 mg/kg thymoquinone improved the number of unilaminar follicles, it remained below the control group (*p* < 0.05). The number of multilaminar and graffian follicles in both thymoquinone treatment groups was the same as in the control group (*p* > 0.05). However, 5 and 10 mg/kg thymoquinone improved the number of antral follicles in PCOS ovaries, it was still below the control group in the 5 mg/kg thymoquinone group (*p* < 0.05). Ovaries of PCOS rats showed a significant increase in the number of atretic follicles compared to the control group (*p* < 0.01), while the number of atretic follicles significantly decreased in PCOS ovaries of rats which received 5 and 10 mg/kg thymoquinone in comparison to PCOS rats (*p* < 0.01). Despite the decrease in the number of atretic follicles in the thymoquinone groups, this value was still higher than the control group (Figures 3 and 4).

### 3.2 The volume of different parts of ovary tissue

**FIGURE 5:**
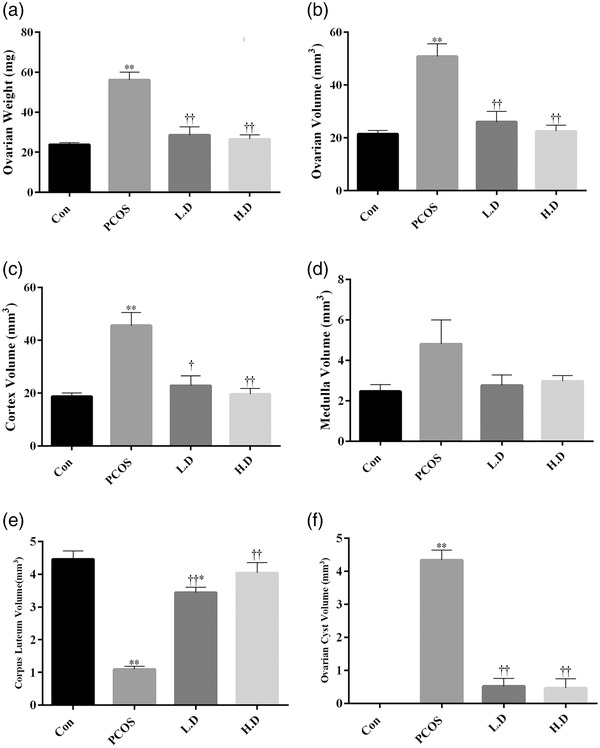
The volume density of cortex, medulla, corpus luteum and ovarian cysts was estimated on 5 μm thickness sections using the point‐counting method (*n* = 8). The results are shown as the Mean ± SD. Abbreviations: Con, control; H.D, PCOS + 10 mg/kg thymoquinone; L.D, PCOS + 5 mg/kg thymoquinone; PCOS, polycystic ovary syndrome. *p < 0.05 and **p < 0.01, Con group vs. PCOS, L.D and H.D. †*p* < 0.05 and ††*p* < 0.01, PCOS vs. L.D and H.D groups.

Ovarian weight and volume, volume density of cortex, medulla, corpus luteum and ovarian cysts were compared between groups. Data showed that PCOS increased the weight and volume of the ovary as well as the volume density of cortex and ovarian cysts when compared to the control group (*p* < 0.01). Both low and high doses of thymoquinone reduced the weight and volume of the ovary, as well as the volume density of the cortex and ovarian cysts in PCOS ovaries (*p* < 0.01), although not significantly different from the control group (*p* > 0.05). PCOS did not have significant negative effects on the volume density of the medulla (*p* > 0.05). Volume density of the corpus luteum was decreased in PCOS (*p* < 0.01) and 5 mg/kg thymoquinone (*p* < 0.05) groups in comparison to the control group (*p* < 0.01). However, administration of both dosages of thymoquinone improved the adverse effect of PCOS on the volume density of corpus luteum (*p* < 0.01), only, 10 mg/kg thymoquinone group had no significant difference for this volume when compared to the control group (Figure 5).

### 3.3 Level of sexual hormones in blood samples

**FIGURE 6:**
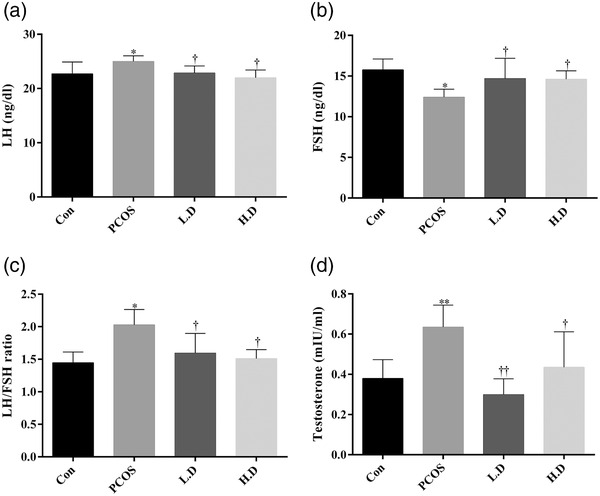
Levels of sexual hormones in blood samples of treatment groups (*n* = 8). The results are shown as the Mean ± SD. Abbreviations: Con, control; H.D, PCOS + 10 mg/kg thymoquinone; L.D, PCOS + 5 mg/kg thymoquinone; PCOS, polycystic ovary syndrome. *p < 0.05 and **p < 0.01, Con group vs. PCOS, L.D and H.D. †*p* < 0.05 and ††*p* < 0.01, PCOS vs. L.D and H.D groups.

Data showed that PCOS increased the levels of LH (*p* < 0.05), LH/FSH ratio (*p* < 0.05) and testosterone (*p* < 0.01) in the blood of female rats when compared to the control group. Administration of thymoquinone in both dosages (5 and 10 mg/kg) significantly decreased the level of LH (*p* < 0.05 and *p* < 0.05), LH/FSH ratio (*p* < 0.05 and *p* < 0.05) and testosterone (*p* < 0.01 and *p* < 0.05) in PCOS groups and caused them to reach to the level of the control group. PCOS, furthermore, led to a depletion in the level of FSH in this group (*p* < 0.05). However, thymoquinone improved this adverse effect of PCOS, and the level of FSH was significantly higher in thymoquinone groups in comparison to the PCOS group (*p* < 0.05, Figure 6).

### 3.4 Gene expression of apoptotic markers and antioxidant enzymes

**FIGURE 7:**
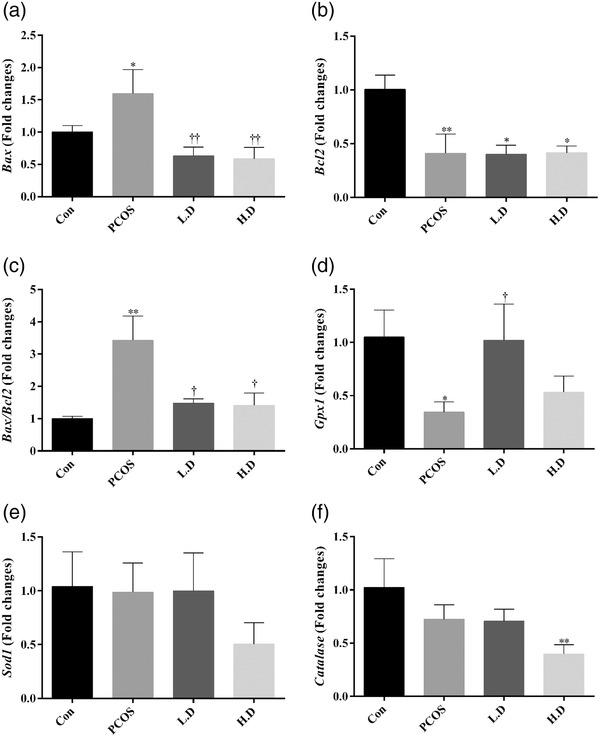
Gene expression of apoptotic markers and antioxidant enzymes. Expression pattern of *Bax* (a), *Bcl2* (b), *Bax/Bcl2* ratio (c), glutathione peroxidase 1 (*GPx1*) (d), superoxide dismutase 1 (*Sod1*) (e) and catalase (*Cat*) (f) in ovarian tissue of different groups. The results are shown as the Mean ± SD. Abbreviations: Con, control; H.D, PCOS + 10 mg/kg thymoquinone; L.D, PCOS + 5 mg/kg thymoquinone; PCOS, polycystic ovary syndrome. *p < 0.05 and **p < 0.01, Con group vs. PCOS, L.D and H.D. †*p* < 0.05 and ††*p* < 0.01, PCOS vs. L.D and H.D groups.

Gene expression of *Bax* (*p* < 0.05) as an apoptotic activator and *Bax/Bcl2* ratio (*p* < 0.01) as an apoptotic marker were significantly upregulated in the ovaries of the PCOS group when compared to the control group. They were markedly decreased in the PCOS groups which received thymoquinone in comparison to the PCOS group (*p* < 0.01 and *p* < 0.05 for *Bax* and *Bax/Bcl2* ratio, respectively). Levels of this gene expression in both thymoquinone groups reach the level of the control group. Significantly lower levels of *Bcl2* transcript were observed in all three PCOS groups when compared to the control group (*p* < 0.05). To investigate if the administration of thymoquinone to PCOS rats changes the expression of three main antioxidant enzymes in ovarian tissue, we quantified the transcripts of glutathione peroxidase 1 (*GPx1*), superoxide dismutase 1 (*Sod1*) and catalase (*Cat*) genes for each group. As shown in Figure 7, transcript levels of the *GPx1* gene were significantly decreased in the ovaries of the PCOS group (*p* < 0.05). However, 5 mg/kg thymoquinone increased the level of *GPx1* gene expression when compared to the PCOS group (*p* < 0.05). The level of *GPx1* gene expression in both thymoquinone groups was not significantly different in comparison to the control group (*p* > 0.05). The result of real‐time RT‐PCR indicated that the relative *Sod1* mRNA expression showed no significant change among groups (*p* > 0.05). The level of gene expression of *catalase* was decreased in the 10 mg/kg thymoquinone group when compared to the control group (*p* < 0.05).

## 4 DISCUSSION

In this study, the protective effects of thymoquinone were studied in a rat model of PCOS. Letrozole as an aromatase inhibitor can prevent the conversion of androgen to oestrogen in the rat ovary, resulting in hyperandrogenism and ovarian polycystic changes (Wang et al., 2020). However, letrozole has been widely used as a rat model for PCOS, it is the most commonly used third‐generation aromatase inhibitor in assisted reproduction. It stimulates ovulation by inhibiting oestrogen secretion; the resulting hypoestrogenic state enhances GnRH release and pituitary FSH production. Thus, letrozole has a beneficial role in anovulatory infertility, PCOS and unexplained infertility (Maladkar et al., 2020). Thymoquinone was utilized at doses of 5 and 10 mg/kg in this investigation. It is a key bioactive component of *N. sativa* that may have medicinal properties, including anti‐inflammatory, anti‐oxidative, anti‐diabetic, anti‐tumour and neuroprotective benefits (Ali & Blunden, 2003; Burits & Bucar, 2000; Hasani‐Ranjbar & Larijani, 2014). We decided to evaluate the effect of thymoquinone in a PCOS rat model because of the relationship between PCOS and folliculogenesis disruption.

Induction of PCOS by letrozole caused a reduction in the number of unilaminar, multilaminar, antral and graffian follicles, and volume density of corpus luteum in ovaries. A significant increase occurred in the number of atretic follicles, weight, and volume of the ovary, volume density of cortex and ovarian cysts in these rats. Gene expression of *Bax* and *Bax/Bcl2* ratio in ovaries, as well as levels of LH, LH/FSH ratio and testosterone in the blood, increased in the PCOS rats. ROS overproduction is closely related to low‐grade inflammation in the ovarian tissues, which may cause hyperandrogenism, such as high levels of testosterone (Kyei et al., 2020). The hypothalamic–pituitary axis generates more LH in response to a high level of androgens (Rosenfield & Ehrmann, 2016). Hyperandrogenism also induces steroidogenic enzymes and leads to the promotion of estradiol, which prevents FHS production (Fuller, 2006). When there is a high level of LH and a low level of FSH, the LH/FSH ratio rises, causing the follicular arrest, atretic follicles, follicular cyst formation, anovulation and a disrupted estrus cycle in PCOS patients (Luchetti et al., 2004). The different developmental stages of folliculogenesis, including the production of unilaminar, multilaminar, antral and graffian follicles, which were disturbed in ovarian tissue of PCOS rats, restored in PCOS rats which received low and high doses of thymoquinone. Also, both doses of thymoquinone could decrease the number of atretic follicles that significantly increased in PCOS rats. This result is in accordance with Shamsi et al., who reported that *licorice* extract had a significant decrease in the number of atretic follicles (Shamsi et al., 2020).

In PCOS, elevated levels of LH and a low concentration of FSH have been documented (Javanshir et al., 2018; McCartney et al., 2002; Nofal et al., 2019). It has been demonstrated that a greater LH level is associated with larger ovarian atretic follicle sizes (Venturoli et al., 1986), which is consistent with our stereological findings. Our previous study on male mice indicated that thymoquinone restored the level of LH and FSH and testosterone in bleomycin‐induced mice (Yaghutian Nezhad et al., 2021). Increasing evidence indicated that plant extracts with antioxidant properties could restore the level of LH in PCOS rates (Nofal et al., 2019; Sadeghi Ataabadi et al., 2017; Sherafatmanesh et al., 2020).

According to our results, administration of thymoquinone could improve symptoms of PCOS and restore normal folliculogenesis in ovaries in a similar way to control rats. PCOS has been linked to an increase in oxidative stress and the generation of ROS. Furthermore, prior investigations have documented that ROS plays a pivotal role in the increase in the number of ovarian atretic follicles, the pathogenesis of ovarian cysts and the reduction in the volume of the corpus luteum (Agarwal et al., 2005; Rezvanfar et al., 2012). Since PCOS exhibits a marked number of cysts in ovarian tissue, therefore, beneficial effects of thymoquinone on PCOS may be due to its antioxidant or antiapoptotic properties. Oxidative stress increases the activity of ovarian steroid‐producing enzymes and induces androgen synthesis, whereas antioxidant compounds prevent their activity (Zhang et al., 2019). A previous study indicated that thylakoid and caraway extracts with antioxidant properties increased the volume of the corpus luteum, and the number of unilaminar, multilaminar, antral and graffian follicles, and decreased the number of atretic follicles (Sherafatmanesh et al., 2020).

Our results showed that the ameliorative influence of thymoquinone is through antioxidant and antiapoptotic mechanisms. Thymoquinone increased the transcript level of the *GPx1* gene to decrease the level of ROS. On the other hand, thymoquinone decreased gene expression of *Bax* as an apoptotic activator and *Bax/Bcl2* ratio as an apoptotic index and improved adverse effects of PCOs. Increased ROS generation can affect mitochondrial activity, resulting in decreased folliculogenesis. The results of some studies showed that antioxidants have positive effects on the management of PCOS. Therefore, studies suggest that using antioxidants is one of the best ways to improve ovarian folliculogenesis in PCOS patients (Johnson et al., 1996; Kugu et al., 1998). Follicular atresia is caused by apoptosis, which is essential for the cyclical growth and regression of follicles in the human ovary (Tilly, 1996). Our data indicated that a reduction in apoptosis happened in the PCOS rats receiving thymoquinone. Thymoquinone acts as a general free radical scavenger as well as a superoxide anion scavenger (Mansour et al., 2002). It seems it is another possible factor that could involve these mechanisms to promote folliculogenesis. Results of an *in‐vitro* study on germinal vesicles derived from PCOS mice indicated that thymoquinone improved maturation, fertilization and blastocyst formation rates. They showed that *Bax* expression was decreased in the matured oocytes treated by 10.0 μM thymoquinone, but *Bcl2* was overexpressed in this group (Eini et al., 2019). Thus, thymoquinone can restore folliculogenesis and sexual hormones by stimulating the antioxidant system and inhibiting the apoptotic pathway, as evidenced by the reduced number of atretic follicles and a larger number of unilaminar, multilaminar, antral and graffian follicles in PCOS‐induced rats given thymoquinone.

In conclusion, our findings show that there are significant variations in the rate of folliculogenesis in PCOS rats which did not receive thymoquinone and those which did. This research adds to our understanding of the molecular mechanisms behind the beneficial effects of thymoquinone on PCOS. Overall, we found that thymoquinone therapy dramatically improved ovarian dysfunction in the PCOS rat model by targeting antiapoptotic and antioxidant capacities. Thus, it might be a safe natural medicine for the treatment of PCOS for clinical usage in humans. However, further studies in rats are needed to assess thymoquinone as a single agent or adjunct treatment when compared with routine PCOS therapy.

## AUTHOR CONTRIBUTIONS

Conceptualization, project administration and supervision: Sanaz Alaee. Data curation, validation and writing – review and editing: Sanaz Alaee and Azizollah Bakhtari. Formal analysis: Farhad Koohpeyma and Azizollah Bakhtari. Investigation and methodology: Maryam Mirani, Zahra Derakhshan, Farhad Koohpeyma and Azizollah Bakhtari. Visualization: Maryam Mirani and Zahra Derakhshan. Writing – original draft: Azizollah Bakhtari.

## CONFLICTS OF INTEREST

The authors declare that there are no conflicts of interest.

### ETHICAL STATEMENT

The study protocol was approved by the Animal Ethical Committee of Shiraz University of Medical Sciences (Ethical code: IR.SUMS.REC.1397.853) and was carried out in accordance with the university's Guideline for the Care and Usage of Laboratory Animals.

### PEER REVIEW

The peer review history for this article is available at https://publons.com/publon/10.1002/vms3.958

## Data Availability

Data are available on request from the authors.
